# Suanzaoren Formulae for Insomnia: Updated Clinical Evidence and Possible Mechanisms

**DOI:** 10.3389/fphar.2018.00076

**Published:** 2018-02-09

**Authors:** Qi-Hui Zhou, Xiao-Li Zhou, Meng-Bei Xu, Ting-Yu Jin, Pei-Qing Rong, Guo-Qing Zheng, Yan Lin

**Affiliations:** Department of Neurology, The Second Affiliated Hospital and Yuying Children's Hospital of Wenzhou Medical University, Wenzhou, China

**Keywords:** suanzaoren, *Semen Ziziphi Spinosae*, Chinese herbal medicine, insomnia, sedative and hypnotic actions

## Abstract

Insomnia disorder is a widespread and refractory disease. *Semen Ziziphi Spinosae*, Suanzaoren, a well-known Chinese herbal medicine, has been used for treating insomnia for thousands of years. Here, we aimed to assess the available evidence of Chinese herbal formulae that contains Suanzaoren (FSZR) for insomnia according to high-quality randomized controlled trials (RCTs) and reviewed their possible mechanisms based on animal-based studies. Electronic searches were performed in eight databases from inception to November 2016. The primary outcome measures were polysomnography index and Pittsburgh sleep quality index. The secondary outcome measures were clinical effective rate and adverse events. The methodological quality of RCTs was assessed by Cochrane's collaboration tool, and only RCTs with positive for 4 out of 7 for the Cochrane risk of bias domains were included in analyses. Thirteen eligible studies with 1,454 patients were identified. Meta-analysis of high-quality RCTs showed that FSZR monotherapy was superior to placebo (*P* < 0.01); FSZR plus Diazepam was superior to Diazepam alone (*P* < 0.05); there were mixed results comparing FSZR with Diazepam (*P* > 0.05 or *P* < 0.05). Furthermore, FSZR caused fewer side effects than that of Diazepam. Suanzaoren contains complex mixtures of phytochemicals including sanjoinine A, Jujuboside A, spinosin and other flavonoids, which has sedative and hypnotic functions primarily mediated by the GABAergic and serotonergic system. In conclusion, the findings of present study supported that FSZR could be an alternative treatment for insomnia in clinic. FSZR exerted sedative and hypnotic actions mainly through the GABAergic and serotonergic system.

## Introduction

Insomnia is characterized by sustained difficulties in initiating or maintaining sleep and cause significant impairment of daytime functioning (American Academy of Sleep Medicine, 2014). Based on the International Classification of Sleep Disorder (ICSD)-3 of the American Academy of Sleep Medicine, chronic insomnia disorder referred to these symptoms that cause clinically significant functional distress or impairment at least three nights per week for at least 3 months, excluding other medical or mental disorders (Morin et al., 2006b). Insomnia is the most common sleep complaint, about one third general population worldwide experiencing insomnia symptoms accompanied by daytime dysfunction consequences and ~50% of patients having a chronic course (Morin et al., 2006b; Buysse, 2013). Persistent insomnia is associated with depression, anxiety disorders, suicide, drug/alcohol abuse, accidents, and cardiovascular disease (Baglioni et al., 2011; Fernandez-Mendoza and Vgontzas, 2013). Moreover, insomnia reduces the quality of life of the patients, and results in increasing healthcare cost and utilization (Leger and Bayon, 2010). At least 90% of insomnia-related costs are resulted from the work absences and reduced productivity (Daley et al., 2009). The treatments of insomnia include pharmacological therapies, psychological and behavioral therapies, and complementary and alternative therapies (CAM) (Krystal, 2009). Benzodiazepines, non-benzodiazepine hypnotics and melatonin receptor agonists are the primary pharmacological therapy. Additionally, the melatonin receptor agonist ramelteon, orexin receptor antagonist suvorexant, and the antidepressant doxepin also have FDA approval for insomnia therapy (Asnis et al., 2015). However, the limited use of these pharmacological treatments is due to the undesirable side-effects such as performance and memory impairment, residual sedation, falls, undesired behaviors during sleep, somatic symptoms, and drug interactions (Wilt et al., 2016). Psychological and behavioral therapies for insomnia are well supported by empirical evidence (Morin et al., 2006a), but they have remained underutilized because of requirement of significant training and long-term implementation (Trauer et al., 2015). Thus, there are rising numbers of insomniac patients who seek to various kinds of CAM around the world.

Traditional Chinese medicine (TCM), as a main part of CAM, includes Chinese herbal medicine (CHM), acupuncture, meditation and massage (Sarris et al., 2011; Zhao, 2013; Liu et al., 2015). A survey in Hong Kong Chinese reported that the most commonly used CAM modalities was CHM (Yeung et al., 2014). CHM formulae (Fufang) are a combination of several CHMs according to TCM theory *Jun-Chen-Zuo-Shi*, known as emperor-miniser-assisstant-courier, first recorded by *Huangdi Neijing* (*Inner Canon of the Yellow Emperor*; Fan et al., 2006). CHM has been used for treating insomnia in China for thousands of years (Li and Deng, 1995), and is still used today, both in China and elsewhere around the world increasingly (Chen et al., 2009; Frass et al., 2012). *Semen ziziphi spinosae*, spine date seed, suanzaoren (SZR), the dried seed of *Ziziphus jujuba* Mill. var. *spinosa* (Bunge) Hu ex H. F. Chou (Huang et al., 2016; Rodríguez and Rodríguez, 2017), is one of the most popular CHMs and has a long history of use in Chinese medicine (Yan, 2010; Yeh et al., 2011; Ni et al., 2015; Rodríguez and Rodríguez, 2017; Shergis et al., 2017; Singh and Zhao, 2017). SZR was first recorded in the *Shennong Bencao Jing* (*Shennong's Classic of Materia Medica*), the earliest medicine monograph of China written 2500 years ago (Gu, 2007). SZR is the most frequently used single herb for treating insomnia (Lei et al., 2015). Systematic reviews of CHM for insomnia have demonstrated that SZR is also the most frequently used herb in randomized controlled trials (RCTs) (Yeung et al., 2012; Ni et al., 2015). Spinosin and jujubosides are the main active compounds of SZR contributing their sedative and hypnotic effects on insomnia (Peng et al., 2000; Li et al., 2003; Jiang et al., 2007). SZR have been widely used in many standard Chinese formulae for insomnia (Li, 2006). In particular, SZR decoction is a well-known classic Chinese herbal formula, and has been used for treating insomnia for more than thousand years, first recorded in *Jingui Yaolue* (*Synopsis of Prescriptions of the Golden Chamber*) by Zhang Zhongjing (AD 152-219). Furthermore, modern pharmacological study indicated that SZR plays an essential role to improve sleep in the SZR decoction (Li and Bi, 2006). However, our previous systematic review indicated that the current evidence is insufficient to support the routine use of SZR decoction for insomnia because of poor methodological quality of the included studies (Xie et al., 2013). In addition, only data from systematic reviews of high-quality RCTs will receive 1a-evidence according to the levels of evidence from the Centre of Evidence-Based Medicine in Oxford (Glasziou et al., 2004). Thus, we conduct an updated systematic review of Chinese herbal formulae that contains SZR (FSZR) for insomnia according to the selected high-quality RCTs.

## Methods

The systematic review and meta-analysis was preformed according to the Preferred Reporting Items in Systematic Reviews and Meta-Analyses (PRISMA) statement (Moher et al., 2010).

### Database and search strategies

We have electronically searched Cochrane Central Register of Controlled Trials (CENTRAL), PubMed, Chinese National Knowledge Infrastructure (CNKI), Wangfang database, Chinese Biomedical Database (CBM), EMBASE and VIP Journals Database from inception to November 2016 by using the following key words: (semen ziziphi spinosae OR ziziphus jujuba OR suan zao ren OR suanzaoren) AND (dyssomnia OR insomnia OR sleep OR sleep disorder OR sleep maintenance OR sleep initiation) AND (randomized controlled trial OR randomized clinical trial OR controlled clinical trial) in English or in Chinese. In addition, we further hand-searched the reference lists from related literature.

### Eligibility criteria

#### Types of studies

RCTs that evaluated the efficacy and safety of FSZR for insomnia were selected, regardless of language, publication status, or population characteristics. Only RCTs with positive for at least 4 out of 7 for the Cochrane risk of bias domains were included in further analyses. Quasi-randomized trials, for example, allocated by medical record number, date of birth, or the order in which participants are included in the study, were excluded.

#### Types of participants

All participants with a diagnosis of insomnia by using Chinese classification of mental disorders (CCMD) (Chinese Society of Psychiatry, 1989, 1995, 2001), or ICSD (American Sleep Disorders Association, 1990; American Academy of Sleep Medicine, 2005, 2014), or Guideline for Clinical Trials of New Patent Chinese Medicines (Zheng, 1993) were included. Insomnia disorder caused by a co-occurring psychiatric or medical condition, or withdrawal from a drug or substance was excluded.

#### Types of interventions

Analyzed interventions in the experimental groups used FSZR, regardless of the dose or the method or the form or the duration. Comparator interventions were given diazepam, placebo or vehicle treatment or basic treatment (i.e., supportive treatment other than diazepam). Studies comparing FSZR with any other TCM agent were excluded.

#### Types of outcome measures

The primary outcome measures were Polysomnography (PSG) index and Pittsburgh Sleep Quality Index (PSQI) (Buysse et al., 1989) at the end of the treatment course. The secondary outcome measures were clinical effective rate and adverse events. Evaluation standards for clinical efficacy based on Guideline for Clinical Trials of New Patent Chinese Medicines (Zheng, 1993) were as follows: (1) clinical recovery: sleep time returned to normal, or the nocturnal sleep time over 6 h, deep sleep, waking up invigorating; (2) markedly effective: significant improvement of sleep, increased at least 3 h of total sleep time; depth of sleep increased; (3) effective: amelioration in symptoms; increased <3 h of total sleep time; (4) ineffective: no significant improvement of sleep, or deteriorated after treatment.

### Study selection and data collection

All studies were searched in the electronic databases independently by two authors. The authors filtered the titles and abstracts of retrieved studies for inclusion. The further review was based on going through the full-text and assessing study eligibility. The exclusion reasons have been recorded. A standardized study extraction was based on first author, population characteristics, study design, study inclusion and exclusion criteria, age and disease duration range of participants, intervention details, duration of treatment, outcomes and follow-up time. For the disagreements, we resolved by discussion between the two authors or hold counsel with the third author.

### Risk of bias in individual studies

The criteria recommended by the Cochrane Collaboration (Higgins and Green, 2011) were used to assess the risk of bias in the included studies. The score ranged from 0 to 7. Divergences were well settled through consulting with correspondence authors.

### Data synthesis and analysis

We performed the statistical analysis on the data by applying the Cochrane Collaboration Review Manager software (RevMan 5.0). Between-study heterogeneity was valued using the chi-square and the *I*^2^ statistic was calculated. The heterogeneity was expected statistically significant if the *P*-values were <0.05. Presence or absence of significant heterogeneity decided the option of random effects model or fixed effects model. Outcome data were calculated using standardized mean difference (SMD) with 95% confidence interval (CI) for continuous outcomes, and risk ratio (RR) with 95% CI for dichotomous outcomes. The Grading of Recommendations Assessment, Development and Evaluation (GRADE) methodology (Schunemann et al., 2013) was used to rate the quality of evidence.

## Results

### Study selection

**Figure 1:**
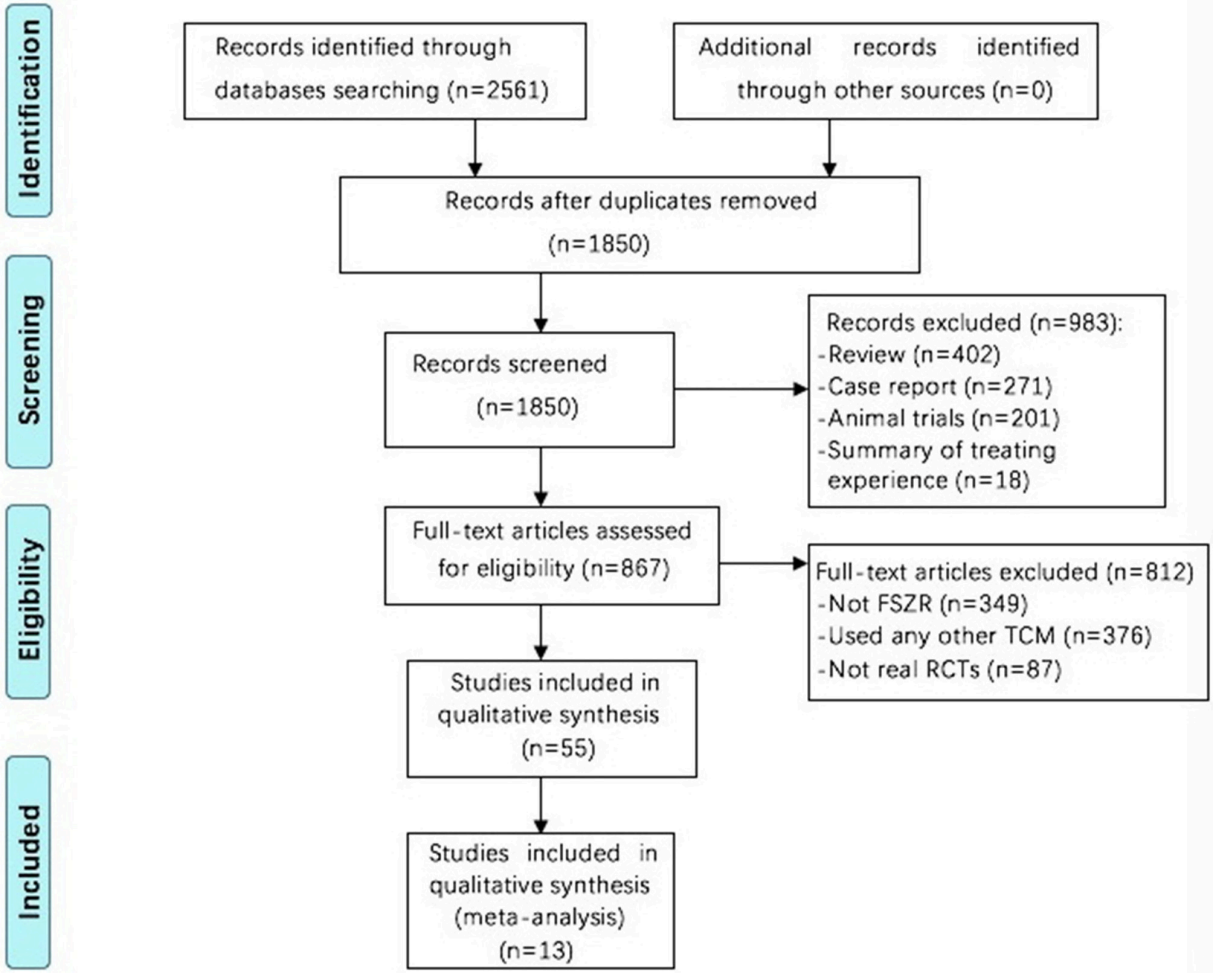
Flowchart of study screening. FSZR, Chinese formulae that contains suanzaoren. TCM, Traditional Chinese medicine; RCTs, randomized controlled trials.

The search strategy has retrieved 2,561 studies in total, among which 711 were considered duplicates. Of the remaining 1,850 articles, 983 articles were eliminated because of review or case report, or summary of treating experience or animal studies. For a further step, we excluded 812 studies by reason that they not used FSZR, or used any other TCM, or not real RCTs. Fifty-five RCTs were left and evaluated by the Cochrane risk of bias tool. Among which, 13 studies (Zhou et al., 2002; Lian et al., 2009; Li et al., 2009; Liu and Nan, 2009; Jiang, 2010; Long, 2010; Wang Z. T. et al., 2010; Jing, 2011; Lu, 2011; Pan, 2011; Wang et al., 2013; Yuan et al., 2013; Shi et al., 2014) were assessed in RCTs with a cumulative score of at least 4 out of 7 for the Cochrane risk of bias tool domains and were ultimately included. The screening process is summarized in a flow diagram (Figure 1).

### Study characteristics

Characteristic of included studies was showed in Table 1. All involved articles were published in Chinese language. All 13 trials were RCTs, and the sample size ranged from 33 to 366, mean aged 26.42 to 71.9 years. The disease durations before treatment lasted from 39 days to 7.9 years. The duration of treatment ranged from 15 days to 12 weeks. Five studies (Zhou et al., 2002; Lian et al., 2009; Jiang, 2010; Yuan et al., 2013; Shi et al., 2014) reported the follow-up time, which lasted from 1 week to 12 weeks. Twelve studies (Lian et al., 2009; Li et al., 2009; Liu and Nan, 2009; Jiang, 2010; Long, 2010; Wang Z. T. et al., 2010; Jing, 2011; Lu, 2011; Pan, 2011; Wang et al., 2013; Yuan et al., 2013; Shi et al., 2014) reported the PSQI score and three studies (Li et al., 2009; Long, 2010; Wang et al., 2013) reported the PSG. The high-frequency herbs in the 13 included articles were detailed in Table 2. The top 6 most frequently used herbs were Spine date seed *(Semen Ziziphi Spinosae*), Indian buead (*Indian buead)*, tuber fleeceflower stem (*Caulis Polygoni Multiflori*), debark peony root (*Radix Paeoniae Alba*), milkwort root (*Radix Polygalae*), Chinese angelica (*Radix Angelicae Sinensis*), which were used more than 3 times. The dose of SZR in each FSZR constituents varied from 24 to 36 g per day (Li et al., 2009; Jiang, 2010; Long, 2010; Pan, 2011). The ingredients and the usage of FSZR formulae were detailed in Table 3. The sleep time was evaluated by Polysomnograghy or electroencephalogram in three studies (Li et al., 2009; Long, 2010; Wang et al., 2013) and PSQI scores in 12 studies (Lian et al., 2009; Li et al., 2009; Liu and Nan, 2009; Jiang, 2010; Long, 2010; Wang Z. T. et al., 2010; Jing, 2011; Lu, 2011; Pan, 2011; Wang et al., 2013; Yuan et al., 2013; Shi et al., 2014). We have detailed the data in the Table 4.

**Table 1:** Characteristics of the included studies.

**Included trials**		**Eligibility criteria**	**Country**	**Study design**	**No. of Participants(female)**		**Mean age (y)**		**Disease duration before treatment**		**Intervention drugs**		**Course of treatment**	**Outcomes**	**Intergroupdifference**	**Follow-up**
					**Experimental**	**Control**	**Experimental**	**Control**	**Experimental**	**Control**	**Experimental**	**Control**				
Shi et al., 2014		CCMD-3	China	RCT	100(45)	100(59)	26.42 ± 2.51	26.84 ± 3.69	15.31 ± 0.85 (m)	14.23 ± 0.59 (m)	TYS	ES	12w	1. PSQI SRSS 2. 5-HIAA; 5-HT; NA 3. Clinical efficacy 4. Adverse events	1. *P* < 0.01 2. *P* < 0.05 3. *P* < 0.05	12w
Yuan et al., 2013		CCMD-3	China	RCT	30(23)	30(26)	39.57 ± 12.38	34.53 ± 11.73	2.18 ± 2.08 (y)	2.18 ± 1.98 (y)	MA+ES	MAplacebo +ES	2w	1. PSQI total score 2. PSQI each factor score 3. Clinical efficacy 4. TCM symptom 5. Adverse events	1. *P* < 0.01 2. *P* < 0.05 3. *P* < 0.01 4. *P* < 0.05	1w
Pan, 2011		GCTNPCM	China	RCT	32(18)	32(14)	39.6 ± 11.1	38.1 ± 9.2	8.9 ± 2.6 (m)	8.2 ± 2.8 (m)	FFAM	ES	4w	1. PSQI 2. Clinical efficacy 3. TCM symptom 4. Sleep improving 5. Adverse events	1. *P* < 0.05 2. N.R. 3. P>0.05 4. P>0.05	N.R.
Jing, 2011		CCMD-3R	China	RCT	21(17)	26(18)	41.82 ± 12.88	42.37 ± 11.64	7.9 ± 3.84 (y)	5.7 ± 2.53 (y)	XSN	Placebo	3w	1. PSQI 2. Clinical efficacy 3. TCM symptom 4. SAS**;** SDS 5. Reducing rate of ES 6. Adverse events	1. *P* < 0.01 2. *P* < 0.05 3. *P* < 0.05 4. *P* > 0.05 5. N.R.	N.R.
Jiang, 2010		CCMD-2-R	China	RCT	183(93)	183(95)	71.9	70.8	33.1 ± 5.7 (m)	31.4 ± 7.1 (m)	FZZR	AP	3w	1. PSQI 2. Clinical efficacy 3. Adverse events	1. *P* < 0.05 2. *P* < 0.05	1m
Long, 2010		CCMD-3	China	RCT	21(N.R.)	21(N.R.)	45.95 ± 10.72	47.57 ± 10.67	N.R.	N.R.	BBZH	Oryzanol	2w	1. PSQI 2. PSG index 3. TCM symptom 4. Adverse events	1. P1 < 0.05 2. N.R. 3. *P* < 0.05	N.R.
Liu and Nan, 2009	TCM vs. WM	ICSD	China	RCT	30(18)	30(17)	36.9 ± 11.48	37.2 ± 11.36	8.9 ± 2.84 (m)	9.3 ± 2.18 (m)	ZRAS	ES	3w	1. PSQI 2. Clinical efficacy	1. *P* < 0.05 2. P>0.05	N.R.
	TCM vs. placebo	ICSD	China	RCT	30(18)	30(17)	36.9 ± 11.48	37.8 ± 11.38	8.9 ± 2.84 (m)	9.8 ± 2.28 (m)	ZRAS	placebo	3w	1. PSQI 3. Clinical efficacy	1. *P* < 0.05 3. *P* < 0.05	N.R.
Li et al., 2009	TCM vs. WM	ICSD-2	China	RCT	9(8)	5(3)	38.4 ± 13.8	37.6 ± 10.3	51.1 ± 32.9 (d)	39.0 ± 37.7 (d)	JWXY+ESplacebo	ES+JWXY placebo	6w	1. PSQI; SRSS 2. PSG index	1. N.R. 2. N.R.	N.R.
	TCM vs. placebo	ICSD-2	China	RCT	9(8)	10(6)	38.4 ± 13.8	30.0 ± 9.7	51.1 ± 32.9 (d)	47.7 ± 33.2 (d)	JWXY +ESplacebo	JWXY placebo +ESplacebo	6w	1. PSQI; SRSS 2. PSG index	1. N.R. 2. N.R.	N.R.
Zhou et al., 2002		CCMD-2-R	China	RCT	58(N.R.)	62(N.R.)	N.R.	N.R.	N.R.	N.R.	SA	ES	15d	1. SDRS 2. HAMA 3. CGI-SI 4. Adverse events	1. *P* < 0.001 2. *P* < 0.05 3. *P* < 0.05	1w
Lian et al., 2009		CCMD-3	China	RCT	71(48)	36(20)	43.14 ± 13.91	44.73 ± 13.92	19.83 ± 27.13 (m)	20.92 ± 45.12 (m)	CYAS	Placebo	3w	1. sleep-score 2. PSQI 3. total sleep time 4. TCM symptom 5. Clinical effective rate 6. Adverse events	1. *P* < 0.01 2. *P* < 0.01 3. *P* < 0.05 4. *P* < 0.05 5. N.R.	1w
Lu, 2011		CCMD-3	China	RCT	36(20)	12(7)	18-65	18-65	1-12(m)	1-12(m)	QXZS	Placebo	4w	1. PSQI 2. Clinical efficacy 3. TCM symptom 4. Adverse event	1. *P* < 0.05 2. *P* < 0.05 3.*P* > 0.05	N.R.
Wang Z. T. et al., 2010		CCMD-3	China	RCT	41(N.R.)	39(N.R.)	N.R.	N.R.	N.R.	N.R.	SQ	Placebo	4w	1. PSQI total score 2. PSQI each factor score 3. Adverse event	1. *P* < 0.05 2. *P* < 0.05	N.R.
Wang et al., 2013		CCMD-3	China	RCT	48(30)	48(32)	45.12 ± 11.51	44.58 ± 12.17	27.16 ± 35.05(m)	25.99 ± 32.17 (m)	ZRAS	Placebo	4w	1. PSQI 2. PSG 3. TCM symptom 4. Adverse event	1. *P* < 0.05 2. *P* < 0.05 3.*P* < 0.05	N.R.

*CCMD, Chinese classification and diagnostic criteria for mental disorders; GCTNPCM, Guideline for Clinical Trials of New Patent Chinese Medicines; ICSD, International classification of sleep disorders; RCT, Randomized controlled trail; N.R., Not reported; y, Year; m, Month; w, Week; d, Day; TYS, Tangyushu powder preparation; ES, Estazolam tablet; MA, Mei'an capsule; FFAM, Fufanganmei decoction; XSN, Xinshenning tablet; FZZR, Fuzhazaoren decoction; AP, Alprazolam tablet; BBZH, Banbaizhenhun decoction; ZRAS, Zaoren'anshen tablet; JWXY, JiaweiXiaoyao powder; SA, Shen'an 3 capsule; CYAS, Chanyeanshen capsule; QXZS, Qingxinzishui capsule; SQ, Sanqi granule; PSQI, Pittsburgh Sleep Quality Index; PSG, Polysomnograghy; SRSS, Self-rating scale of sleep; TCM, Traditional Chinese medicine; SAS, Self-rating depression scale; SDS, Self-rating anxiety scale; SDRS, Sleep dysfunction rating scale; HAMA, Hamilton anxiety scale; CGI, Clinical general impression scale; P, The significant difference between experimental and control group*.

**Table 2:** Analysis of the high frequency herbs in treatment of insomnia.

**Chinese name**	**Common name**	**Latin name**	**Frequency**
Suanzaoren	Spine date seed	*Semen ZiziphiSpinosae*	13
Fuling	Indian buead	*Indian buead*	4
Shouwuteng	tuber fleeceflower stem	*Caulis PolygoniMultiflori*	4
Baishao	debark peony root	*Radix Paeoniae Alba*	4
Yuanzhi	milkwort root	*Radix Polygalae*	3
Danggui	Chinese angelica	*Radix AngelicaeSinensis*	3
Shichangpu	grassleafsweetflag rhizome	*RhizomaAcoriTatarinowii*	2
Baihe	lily bulb	*BulbusLilii*	2
Chaihu	Chinese thorowax root	*Radix Bupleuri*	2
Huanglian	golden thread	*RhizomaCoptidis*	2
Zhenzhumu	nacre	*Concha Margaritifera*	2
Banxia	pinellia tuber	*RhizomaPinelliae*	2
Longchi	Dragon's Teeth	*Mastodifossiliadentis*	2
Chenpi	dried tangerine peel	*PericarpiumCitriReticulatae*	2

**Table 3:** Ingredients and usage of FSZR formulae.

**Included studies**	**Prescription**	**Constitution**	**Usage**	**Preparations**
Shi et al., 2014	TYS	Semen ZiziphiSpinosae, RhizomaCoptidis, PericarpiumCitriReticulatae, Caulis Bambusae in Taenia, RhizomaAcoriTatarinowii, Radix Polygalaea	15 g tidpo	Power
Yuan et al., 2013	MA+ES	Semen ZiziphiSpinosae, Radix Ginseng, Eleutherococcussenticosus, Indian buead, Radix AngelicaeSinensis, RhizomaLigusticiChuanxionga	4 g qnpo	Granule
Pan, 2011	FFAM	Semen ZiziphiSpinosae 12 g, OsDraconis 9 g, Concha Margaritifera 9 g, Caulis PolygoniMultiflori 12 g, FructusSchisandraeChinensis 3 g, Radix Paeoniae Alba 9 g, Radix RehmanniaeRecens 9 g	One dose bid po	Decoction
Jing, 2011	XSN	Semen ZiziphiSpinosae, Indian buead, Caulis PolygoniMultiflori, Massa MedicataFermentata, FructusGardeniaea	4 g tidpo	Tablet
Jiang, 2010	FZZR	Semen ZiziphiSpinosae 15 g, Indian buead 12 g, FructusCrataegi 15 g	One dose bid po	Decoction
Long, 2010	BBZH	Semen ZiziphiSpinosae 30 g, RhizomaPinelliae 10 g, BulbusLilii30 g, Radix Curcumae 10 g, Caulis PolygoniMultiflori 30 g, Magnetitum 30 g, Concha Ostreae 30 g, Mastodifossiliadentis 30 g, Concha Margaritifera 30 g, Indian buead 15 g, PericarpiumCitriReticulatae 10 g, RhizomaAcoriTatarinowii 10 g	Half dose bid po	Decoction
Liu and Nan, 2009	ZRAS	Semen ZiziphiSpinosae, Radix SalviaeMiltiorrhizae, FructusSchisandraeChinensisa	5 # qnpo	Tablet
Li et al., 2009	JWXY	Semen ZiziphiSpinosae 18 g, Poria cum Radix Pini 15 g, Radix Bupleuri 9 g, Radix AngelicaeSinensis 10 g, Radix Paeoniae Alba 15 g, RhizomaAtractylodisMacrocephalae 12 g, Radix Glycyrrhizae 5 g, HerbaMenthae 5 g, RhizomaZingiberisRecens 4 g	250ml bid po	Power
Zhou et al., 2002	SA	Ganoderma, BulbusLilii, Radix PaeoniaeRubra, Radix SalviaeMiltiorrhizae, Cortex MoutanRadicis, Semen ZiziphiSpinosae, Radix Pseudostellariae, Mastodifossiliadentis, Succinuma	5 # qnpo	Capsule
Lian et al., 2009	CYAS	Semen ZiziphiSpinosae, Periostracum Cicadae, Caulis PolygoniMultiflori, BombyxBatryticatus, Lumbricus, Radix Paeoniae Alba, RamulusUncariae Cum Uncis, RhizomaPinelliae, Radix Polygalaea	5# qnpo	Capsule
Lu, 2011	QXZS	Semen ZiziphiSpinosaea, RhizomaCoptidis, Radix Scutellariae, Radix Paeoniae Alba, CollaCoriiAsini, Cortex Cinnamomi, Cortex Albiziae	5 # tidpo	Capsule
Wang Z. T. et al., 2010	SQ	Semen ZiziphiSpinosae, Caulis Spatholobi, Radix Notoginseng, Cirsiumjaponicuma	One dose qdpo	Granule
Wang et al., 2013	ZRAS	Semen ZiziphiSpinosae, Caulis PolygoniMultiflori, Fructus Mori, FlosAlbiziae, Semen Platycladi, Radix AngelicaeSinensis, Radix RehmanniaePreparata, Radix Polygalae, Radix Bupleuria	4 # tidpo	Tablet

*TYS, Tangyushu powder preparation; MA, Mei'ancapsule; FFAM, Fufanganmeidecoction; XSN, Xinshenning tablet; FZZR, Fuzhazaoren decoction; BBZH, Banbaizhenhun decoction; ZRAS, Zaoren'anshen tablet; JWXY, JiaweiXiaoyao powder; SA, Shen'an 3 capsule; CYAS, Chanyeanshen capsule; QXZS, Qingxinzishui capsule; SQ, Sanqi granule; bid, bis in die; d:day; po, peros; qd, quaquedie; tid, ter in die; w, week; #, tablet*.

**Table 4:** Characteristics of the Sleeping time of included studies.

**Included trials**		**Methods**	**Sleeping-time (before)**		**Sleeping-time (after)**	
			**Experimental**	**Control**	**Experimental**	**Control**
Shi et al., 2014		PSQI	NG	NG	NG	NG
Yuan et al., 2013		PSQI	1.53 ± 1.07	1.53 ± 0.97	1.10 ± 0.88	1.33 ± 0.96
Pan, 2011		PSQI	2.58 ± 1.01	2.46 ± 0.93	1.05 ± 0.99	1.71 ± 0.86
Jing, 2011		PSQI	NG	NG	NG	NG
Jiang, 2010		PSQI	2.7 ± 0.5	2.7 ± 0.7	0.6 ± 0.7	1.2 ± 0.7
Long, 2010		PSG(m)	360.60 ± 32.975	355.76 ± 30.363	368.07 ± 32.794	358.71 ± 34.229
Liu and Nan, 2009	TCM vs. WM	PSQI	NG	NG	NG	NG
	TCM vs. placebo	PSQI	NG	NG	NG	NG
Li et al., 2009	TCM vs. WM	PSG(m) PSQI	367.5 ± 52.0 NG	405.7 ± 38.2 NG	391.8 ± 44.5 NG	378.6 ± 53.9 NG
	TCM vs. placebo	PSG(m) PSQI	367.5 ± 52.0 NG	385.8 ± 43.4 NG	391.8 ± 44.5 NG	405.6 ± 49.4 NG
Zhou et al., 2002		PSQI	NG	NG	NG	NG
Lian et al., 2009		PSQI	4.91 ± 0.87	4.55 ± 1.06	6.29 ± 1.17	5.22 ± 1.16
Lu, 2011		PSQI	2.39 ± 0.68	2.49 ± 0.69	1.12 ± 0.67	2.32 ± 0.72
Wang Z. T. et al., 2010		PSQI	4.32 ± 1.56	4.18 ± 1.81	5.77 ± 1.47	4.87 ± 1.99
Wang et al., 2013		PSG(m) PSQI	339.66 ± 77.1 NG	368.90 ± 70.12 NG	388.26 ± 74 NG	367.93 ± 86.07 NG

*N.G., Not given; w, Week; m, minite; TYS, Tangyushu powder preparation; ES, Estazolam tablet; MA, Mei'an capsule; FFAM, Fufanganmei decoction; XSN, Xinshenning tablet; FZZR, Fuzhazaoren decoction; AP, Alprazolam tablet; BBZH, Banbaizhenhun decoction; ZRAS, Zaoren'anshen tablet; JWXY, JiaweiXiaoyao powder; SA, Shen'an 3 capsule; CYAS, Chanyeanshen capsule; QXZS, Qingxinzishui capsule; SQ, Sanqi granule; PSQI, Pittsburgh Sleep Quality Index; PSG, Polysomnograghy; P, The significant difference between experimental and control group*.

### Risk of bias within studies

The Cochrane's risk of bias score of included studies ranged from 4 to 7 (Table 5). All of the retrieved articles belonged to RCT. Among which, 11 out of 13 articles described the method of random sequences generation. Four studies (Lian et al., 2009; Li et al., 2009; Pan, 2011; Yuan et al., 2013) implemented the concealment allocation. The blinding was used in 11 studies except two studies (Pan, 2011; Shi et al., 2014), including seven double blinding (Zhou et al., 2002; Lian et al., 2009; Li et al., 2009; Wang Z. T. et al., 2010; Jing, 2011; Wang et al., 2013; Yuan et al., 2013) and four single blinding (Liu and Nan, 2009; Jiang, 2010; Long, 2010; Lu, 2011). There were five studies (Lian et al., 2009; Li et al., 2009; Jing, 2011; Wang et al., 2013; Yuan et al., 2013) described the blinding procedure. Three studies (Li et al., 2009; Yuan et al., 2013; Shi et al., 2014) avoided the detection bias by blind the statisticians. Selection bias and other bias were not found in all included studies.

**Table 5:** The methodological quality of included studies.

	**A**	**B**	**C**	**D**	**E**	**F**	**G**	**Total score**
Shi et al., 2014	+	?	?	+	+	+	+	5+
Yuan et al., 2013	+	+	+	+	+	+	+	7+
Pan, 2011	+	+	?	+	?	+	+	5+
Jing, 2011	+	?	+	+	?	+	+	5+
Jiang, 2010	+	?	+	+	?	+	+	5+
Long, 2010	+	?	+	+	?	+	+	5+
Liu and Nan, 2009	+	?	+	+	?	+	+	5+
Li et al., 2009	+	+	+	+	+	+	+	7+
Zhou et al., 2002	+	?	+	+	?	+	+	5+
Lian et al., 2009	+	+	+	+	?	+	+	6+
Lu, 2011	?	?	+	+	?	+	+	4+
Wang Z. T. et al., 2010	+	?	+	+	?	+	+	5+
Wang et al., 2013	?	?	+	+	?	+	+	4+

*A, random sequence generation; B, allocation concealment; C, blinding of participants and personnel; D, blinding of outcome assessment; E, imcomplete outcome data; F, selective reporting; G, other sources of bias*.

### Effectiveness

#### FSZR vs. placebo

**Figure 2:**
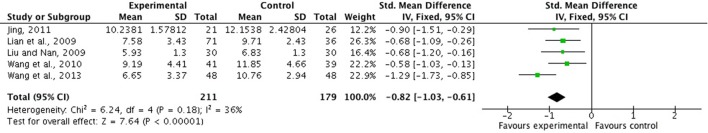
PSQI scores of FSZR vs. placebo. PSQI, Pittsburgh Sleep Quality Index; FSZR, Chinese formulae that contains suanzaoren.

**Figure 3:**
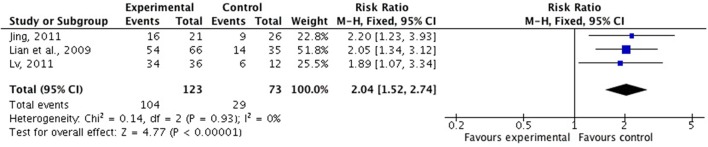
Clinical effective rate of FSZR vs. placebo. FSZR, Chinese formulae that contains suanzaoren.

There were six studies (Lian et al., 2009; Liu and Nan, 2009; Wang Z. T. et al., 2010; Jing, 2011; Lu, 2011; Wang et al., 2013) comparing FSZR with placebo for treating insomnia (Table 1). Meta-analysis of above six studies showed significant between-group difference in PSQI scores (*n* = 438, SMD = −1.05, 95% CI: −1.49 to −0.60, *P* < 0.00001, heterogeneity X^2^ = 23.36, df = 5, *P* = 0.004, *I*^2^ = 74%). After removing study by Lu et al. (Lu, 2011) that was considered the potential sources of the heterogeneity because of small sample size, meta-analysis of five studies (Lian et al., 2009; Liu and Nan, 2009; Wang Z. T. et al., 2010; Jing, 2011; Wang et al., 2013) showed that FSZR better reduce the PSQI score than that of placebo in PSQI scores (*n* = 390, SMD = −0.82, 95% CI: −1.03 to −0.61, *P* < 0.00001, heterogeneity X^2^ = 6.24, df = 4, *P* = 0.18, *I*^2^ = 36%; Figure 2). Meta-analysis of four studies (Lian et al., 2009; Liu and Nan, 2009; Jing, 2011; Lu, 2011) found significant difference in clinical effective rate between the FSZR and placebo groups (*n* = 256, RR: 1.73, 95% CI 1.25 to 2.39, *P* = 0.0009, heterogeneity X^2^ = 6.59, df = 3, *P* = 0.09, *I*^2^ = 54%). After removing study by Liu et al. (Liu and Nan, 2009) whose diagnostic criteria differed from other three studies, meta-analysis of three studies (Lian et al., 2009; Jing, 2011; Lu, 2011) showed that FSZR was significantly more effective than that of placebo in clinical effective rate (*n* = 196, RR: 2.04, 95% CI 1.52 to 2.74, *P* < 0.00001, heterogeneity X^2^ = 0.14, df = 2, *P* = 0.93, *I*^2^ = 0%; Figure 3).

#### FSZR vs. diazepam

Five studies (Zhou et al., 2002; Liu and Nan, 2009; Jiang, 2010; Pan, 2011; Shi et al., 2014) investigated FSZR vs. Diazepam. The meta-analysis was not conducted because of high heterogeneity. Three studies (Jiang, 2010; Pan, 2011; Shi et al., 2014) found that FSZR was more effective than Diazepam in PSQI score (*P* > 0.05), whereas one study (Liu and Nan, 2009) showed no significant difference. FSZR was significantly improving clinical effective rate in two studies (Jiang, 2010; Shi et al., 2014) (*P* < 0.05), but not in other two studies (Liu and Nan, 2009; Pan, 2011) (*P* > 0.05) relative to Diazepam.

#### FSZR plus diazepam vs. FSZR placebo plus diazepam

**Figure 4:**
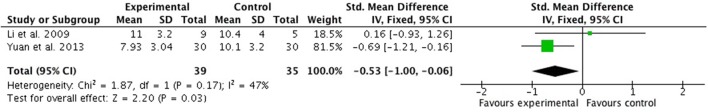
PSQI scores of FSZR plus Diazepam vs. FSZR placebo plus Diazepam. PSQI, Pittsburgh Sleep Quality Index; FSZR, Chinese formulae that contains suanzaoren.

Two studies (Li et al., 2009; Yuan et al., 2013) compared FSZR plus Diazepam with FSZR placebo plus Diazepam. Meta-analysis showed a significant reduction in PSQI scores for the combination therapy relative to estazolam alone (*n* = 74, SMD = −0.53, 95% CI: −1.00 to −0.06, *P* = 0.03, heterogeneity X^2^ = 1.87, df = 1, *P* = 0.17, *I*^2^ = 47%) (Figure 4). The study by Yuan et al. (2013) found FSZR was significantly more effective than placebo in clinical effective rate (*P* < 0.05).

### Adverse events

Twelve out of 13 studies reported adverse events. One study (Shi et al., 2014) reported adverse events in both experimental group and control group, including acratia, somnolence, dizzy, diarrhea, and dry mouth. Two studies (Zhou et al., 2002; Jiang, 2010) reported side effects in control group alone, including dry mouth, constipation, nausea, somnolence, dizzy, fatigued, and memory decline. Serious and life-threatening adverse events, such as an irregular heartbeat, seizures, and death were not happened in all included studies.

### Mechanisms of SZR for insomnia

**Figure 5:**
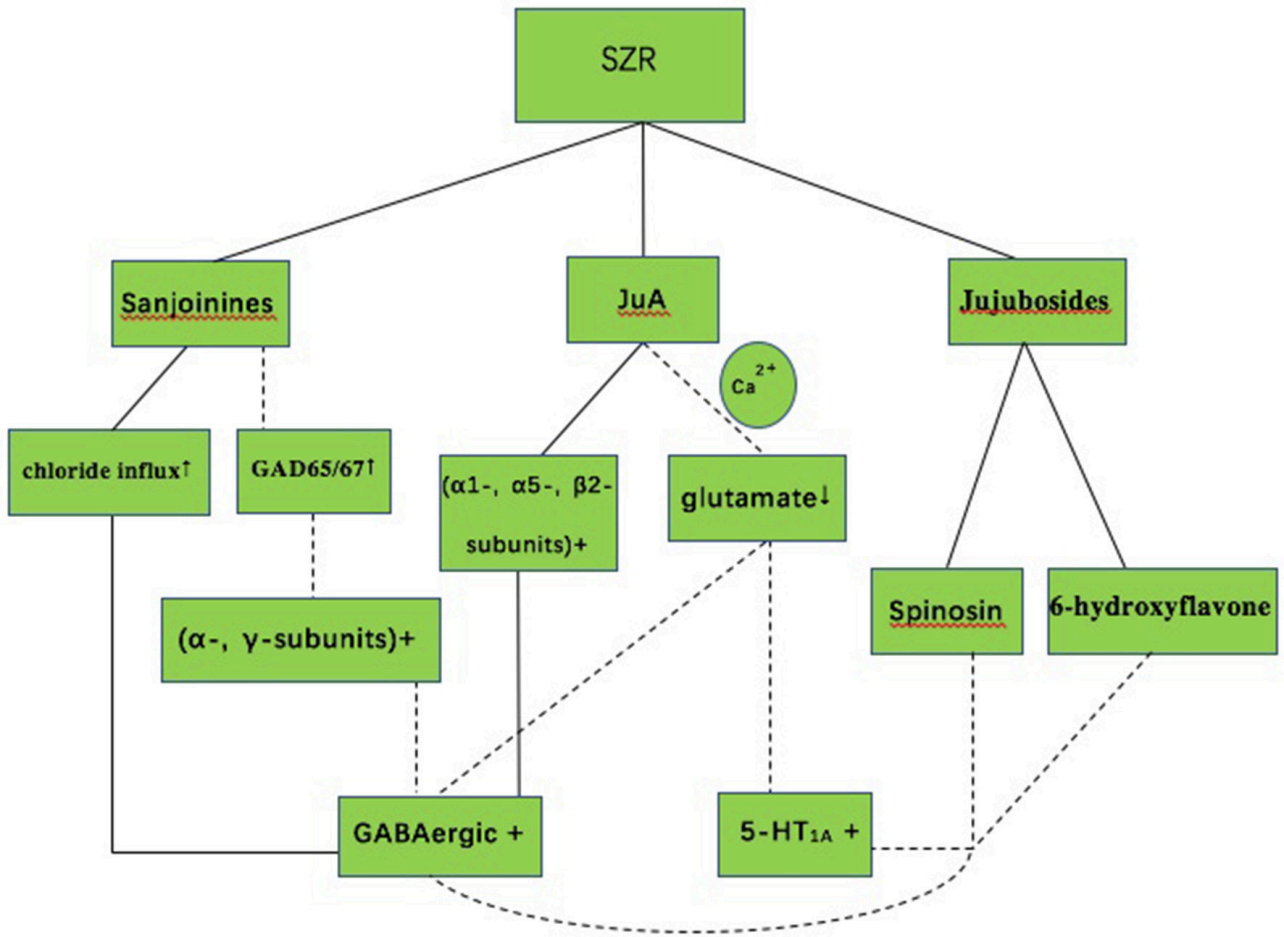
The mechanism of sedative-hypnotic effect in SZR for insomnia. SZR, suanzaoren; GABA, gamma-aminobutyric acid; 5-HT_1A_, 5-hydroxytryptamine (1A); JuA, Jujuboside A; Solid lines indicate established effects, whereas dashed lines represent putative mechanism.

SZR exerts a range of sedative and hypnotic actions mediated primarily by the GABAergic and serotonergic system (Ma et al., 2007; Wang L. E. et al., 2010; Shergis et al., 2017) (Figure 5). The SZR typically contains complex mixtures of phytochemicals, including sanjoinine A, Jujuboside A (JuA), spinosin and other flavonoids (Yang et al., 2013). Sanjoinine A, one of the aporphine alkaloid from SZR, was demonstrated to enhance sleep behaviors and augment pentobarbital-induced sleeping behaviors through GABAergic system (Han et al., 2009; Rodríguez and Rodríguez, 2017). Furthermore, Sanjoinine A increases chloride influx and GABA synthesis via glutamic acid decarboxylase (GAD 65/67) activation in cultured cerebellar granule cells, inducing prolonged sleeping time (Zhang et al., 2003). JuA inhibits the rat hippocampus excitatory state *in vivo* and *in vitro* through glutamate-mediated excitatory signal pathway (Cao et al., 2010), affecting GABAergic and serotonergic system. Low dose of JuA exerts sedative-hypnotic effects related to increasing the GABAA receptor (α1, α5, ß2) gene expression (You et al., 2010). Flavonoids in the water extract of SZR also contribute to insomnia treatment. The flavonoid 6-hydroxyflavone is involved in the binding of GABAA receptors showing partial agonistic action (Ren et al., 2010). The administration of spinosin, a C-glycoside flavonoid, showed significant sedative effects at increasing total sleep time and reducing sleep latency in pentobarbital treated rat group (Lee et al., 2016).

### The quality of the evidence

Summary of Findings (SOF) tables were provided by the GRADE profiler. The quality of the evidence was high or moderate according to the GRADE assessment, we summarized the result in Table 6.

**Table 6:** Quality of evidence by GRADE system.

**Quality assessment**		**Risk of bias**	**Inconsistency**	**Indirectness**	**Imprecision**	**Other considerations**	**No. of patients**		**Quality**	**Importance**
**No. of studies**	**Design**						**FSZR**	**Placebo**		
**PSQI (FOLLOW-UP MEAN 3.4 WEEKS; BETTER INDICATED BY LOWER VALUES)**										
5	RCTs	Serious	No serious	No serious	No serious	none	211	179	Moderate	Critical
			inconsistency	indirectness	imprecision					
**CLINICAL EFFECTIVE RATE (FOLLOW-UP MEAN 3.3 WEEKS)**										
3	RCTs	Serious	No serious	No serious	No serious	Strong	128	74	High	Critical
			inconsistency	indirectness	imprecision	association				

*RCT: randomized controlled trial; PSQI, Pittsburgh Sleep Quality Index; FSZR, Chinese formulaethat contains Suanzaoren*.

## Discussion

### Summary of main findings

Our previous system review (Xie et al., 2013) showed that the current evidence was insufficient to support the efficacy and safety of SZRD for insomnia due to lack of high-quality RCTs. The present study is an updated systematic review based on the high-quality RCTs. Thirteen studies with 1,454 individuals were identified in analysis. The findings demonstrated that FSZR used as a monotherapy was superior to placebo and as an adjunct therapy was superior to Diazepam alone in terms of PSQI score and clinical effective rate, whereas there were mixed results comparing FSZR with Diazepam directly. There were fewer adverse effects in comparison with controls and no life-threatening side effects were happened in all included studies. The quality of the evidence was high or moderate based on the updated GRADE methodology and profiler.

## Limitations

The strength of this study was that all studies included had low risk of bias with positive for at least four out of seven for the Cochrane risk of bias domains. However, we did acknowledge that there were some methodological limitations which were worth noting. Firstly, allocation concealment was used in two studies (Ma et al., 2007; Moher et al., 2010). The trials with inadequate or unclear concealment of allocation were about 18% more “beneficial” than that with adequate concealment (95% CI 5% to 29%; Higgins and Green, 2011). Secondly, blinding was an essential method to limit the occurrence of performance bias and ascertainment bias in clinical trials (Health Canada, 2003). Although there were 11 out of 13 studies that mentioned the blinding, double-blinding and placebo design was reported in six studies (Lian et al., 2009; Wang Z. T. et al., 2010; Jing, 2011; Lu, 2011; Wang et al., 2013; Yuan et al., 2013). One of the main reasons for not using double-blinded study was that the placebos of CHMs is difficult to prepared in the same color, flavor and taste. Thirdly, the follow-up period was not long enough to achieve crucial results because insomnia may wax and wane with or without treatment. Thus, a longer follow-up period was necessary (Xie et al., 2013). At least 6-month follow-up may assess whether a sustained effect and safety of FSZR for insomnia can persist for a long period (European Medicines Agency, 2011). Finally, the quality control of herbal preparations is crucial for the validity of the study results. However, only some patent FSZR mentioned in this important issue. Suanzaoren contains complex mixtures of phytochemicals such as sanjoinine A, Jujuboside A, spinosin, and other flavonoids, which can be further used for the marker of quality control of the herbal preparations.

### Implication for practice

This is an updated system review of high-quality RCTs to assess the efficacy and safety of FSZR for insomnia. In the present study, patients receiving FSZR monotherapy was superior to placebo, and equivalent to Diazepam. Combined FSZR with Diazepam was superior to Diazepam alone. Furthermore, FSZR caused fewer side effects than that of Diazepam. Therefore, FSZR therapy may be effective and well tolerated for the treatment of insomnia. Taken together, our findings supported that clinicians may option FSZR as an alternative treatment for insomnia.

### Implication for research

There are many implications arising from research. First, although double-blinding is encouraged in RCTs, it is inherently difficult in herbal placebo because of the specific color, flavor, and taste of CHMs (Ni et al., 2015). One of possible solutions is that herbs can be prepared as tablets or capsules to facilitate the development of a convincing placebo (Fu et al., 2013); however, the use of capsules is still challenged because of the change of efficacy and characteristics of drugs (Stegemann and Bornem, 1999). Second, SZR is the most frequently used herb for insomnia, and it is regarded as an essential constitute in numbers of classical herbal formulae (Li and Bi, 2006; Lei et al., 2015). In the present study, the most frequently used herbs were *Semen Ziziphi Spinosae, Indian buead, Caulis Polygoni Multiflori, Radix Paeoniae Alba, Radix Polygalae*, Radix Angelicae Sinensis, which is worth further carrying out rigor RCTs as candidate formula. Third, Sanjoinine A, JuA, and flavonoids in the water extract of SZR contributed to its sedative-hypnotic effects. The increasing of chloride influx and over-expression of α- and γ-subunit GABA receptor was involved in the mechanisms of these effects. The modification of serotonin and glutamate also inducing sleep, but the exact role of the regulation of sleep is still unknown. Further experimental studies are required to unravel the mechanisms of FSZR for insomnia.

## Conclusion

In the present study, the findings demonstrated that FSZR therapy was effective and well tolerated for insomnia through sedative and hypnotic actions primarily mediated by the GABAergic and serotonergic system. Thus, FSZR could be an alternative treatment for insomnia in clinical practice.

## Author contributions

G-QZ and YL: contribute as the senior authors and the principal investigator (PI) of this study; Q-HZ, X-LZ, and M-BX: write the first draft of the manuscript and contribute to the overall design; G-QZ and YL: refine the study; T-YJ and P-QR: identified, reviewed studies for eligibility and performed the meta-analysis of data; All authors read, critically reviewed and approved the final manuscript.

### Conflict of interest statement

The authors declare that the research was conducted in the absence of any commercial or financial relationships that could be construed as a potential conflict of interest.
